# ATM-Deficient Cancers Provide New Opportunities for Precision Oncology

**DOI:** 10.3390/cancers12030687

**Published:** 2020-03-14

**Authors:** Nicholas R. Jette, Mehul Kumar, Suraj Radhamani, Greydon Arthur, Siddhartha Goutam, Steven Yip, Michael Kolinsky, Gareth J. Williams, Pinaki Bose, Susan P. Lees-Miller

**Affiliations:** 1 Department of Biochemistry and Molecular Biology, Robson DNA Science Centre, Charbonneau Cancer Institute, Cumming School of Medicine, University of Calgary, 3330 Hospital Drive NW, Calgary, AB T2N 1N4, Canada; nrjette@ucalgary.ca (N.R.J.); mehul.kumar@ucalgary.ca (M.K.); suraj.radhamani@ucalgary.ca (S.R.); greydon.arthur@ucalgary.ca (G.A.); goutam@ualberta.ca (S.G.); gareth.williams2@ucalgary.ca (G.J.W.); pbose@ucalgary.ca (P.B.); 2 Tom Baker Cancer Centre, 1331 29 St NW, Calgary, AB T2N 4N2, Canada; smyip@ualberta.ca; 3 Cross Cancer Institute, 11560 University Avenue NW, Edmonton, AB T6G 1Z2, Canada; michael.kolinsky@albertahealthservices.ca

**Keywords:** ATM, olaparib, ATR, PARP, PARP inhibitor, prostate cancer, pancreatic cancer, lung cancer

## Abstract

Poly-ADP ribose polymerase (PARP) inhibitors are currently used in the treatment of several cancers carrying mutations in the breast and ovarian cancer susceptibility genes *BRCA1* and *BRCA2*, with many more potential applications under study and in clinical trials. Here, we discuss the potential for extending PARP inhibitor therapies to tumours with deficiencies in the DNA damage-activated protein kinase, Ataxia-Telangiectasia Mutated (ATM). We highlight our recent findings that PARP inhibition alone is cytostatic but not cytotoxic in ATM-deficient cancer cells and that the combination of a PARP inhibitor with an ATR (ATM, Rad3-related) inhibitor is required to induce cell death.

## 1. PARP and PARP Inhibitors

Genome instability, characterized by the accumulation of mutations and chromosomal alterations in the genome, is both a hallmark and a driver of cancer [1,2]. Yet the same genomic alterations that predispose a cell to cancer may also render cells susceptible to targeted therapies. Accordingly, a goal of precision oncology is to achieve better cancer control by targeting therapy to specific genetic defects or aberrations in the tumour, while causing less damage to normal tissue and consequently, fewer side-effects. One of the most dramatic examples of success in this area has been the use of poly-ADP-ribose polymerase (PARP) inhibitors in the treatment of patients with tumours that harbour inactivating mutations in the breast and ovarian cancer susceptibility genes, *BRCA1* and *BRCA2*.

PARP was identified in the 1960s as an enzyme that metabolizes nicotinamide adenine dinucleotide, NAD^+^ [3]. Early studies indicated that inhibition of PARP blocked repair of DNA strand breaks and PARP inhibitors were soon considered as potential radiation sensitizers [4]. The first PARP inhibitor, a simple analogue of nicotinamide was generated in 1971 [5] and, given the reported roles of PARP in cell death and ischemia as well as DNA repair, there was increasing interest in the clinical applications of PARP inhibition [6,7]. PARP is now established as one of a family of poly-ADP polymerases of which PARPs 1, 2 and 3 have roles in the DNA damage response [8]. Of these, PARP 1 is the most well-studied and the one to which we will refer in this review. PARP1 binds avidly to ends of DNA that occur at single and double DNA strand breaks and then, in a process called PARylation, auto-modifies to create long polymers of poly-ADP-ribose (PAR). PAR chains interact with proteins involved in DNA repair and other pathways, recruiting them to sites of DNA damage [9]. Importantly, PARylation also serves to disengage PARP from DNA ends [10]. Most PARP inhibitors in use today prevent PARylation, leading to trapping of PARP at DNA ends [11]. It was originally proposed that PARP inhibition blocked base excision repair and single strand break repair pathways thus increasing reliance on BRCA-dependent repair. However, this model has been challenged [12] and more recent studies have shown that olaparib reduces cell proliferation by inducing replication stress [13] and that olaparib sensitivity is due to engagement of homologous recombination repair (HRR) at replication forks [14].

In 2005, two seminal papers were published demonstrating that breast cancer cells with siRNA depletion of BRCA1 or BRCA2 were exquisitely sensitive to the PARP inhibitor NU1025 [15,16]. *BRCA1* and *BRCA2* are cancer predisposition genes that are inactivated in ~25% of inherited breast cancers, ~15% of all ovarian cancers and several other cancers, suggesting that PARP inhibitors might have potential in treating a wide-range of patients with BRCA-deficient tumours [17]. The PARP inhibitor AZD2881, also known as olaparib or Lynparza^TM^, showed promise in mouse models of breast cancer [18] and quickly moved into clinical trials, showing anti-tumour activity in BRCA-mutated cancers, even in phase I studies [19]. Olaparib, the first PARP inhibitor to gain regulatory approval, is now FDA-approved in advanced ovarian [20], breast [21], pancreatic [22] and prostate cancers [23], with the PARP inhibitors rucaparib [24], niraparib [25] and talazoparib [26] also FDA-approved in varying indications [17,27].

BRCA1 and 2 play critical roles in detection, signalling and repair of DNA double strand breaks (DSBs) via the HRR pathway. HRR is active in S phase at stalled replication forks and in G2 phase of the cell cycle after DSBs have been resected to contain long ssDNA overhangs on their 3’ ends [28]. These long regions of ssDNA are bound by replication protein A (RPA) and BRCA2 plays a role in the replacement of RPA with RAD51, the protein that initiates strand invasion and the search for a homologous DNA sequence during HRR [28]. BRCA1 interacts with BRCA2 via the PALB2 protein, and is recruited to DNA damage-induced foci where it participates in activating DNA repair and cell signalling pathways [29]. Given the encouraging early results showing PARP inhibitor sensitivity in BRCA-deficient cells, screens were initiated to identify other proteins that when knocked down with siRNA might confer sensitivity to PARP inhibitors [30,31,32]. One of these was Ataxia Telangiectasia Mutated (ATM).

## 2. ATM

ATM is a member of the phosphatidylinositol-3 kinase-like (PIKK) family of serine/threonine protein kinases with critical roles in the cellular response to DNA damaging agents, such as ionizing radiation (IR), that produce DSBs [33]. Like other members of the PIKK family, ATM is a large protein of over 350 kDa that is composed of an extended N-terminal region containing multiple HEAT (Huntingtin, Elongation factor 3, A subunit of protein phosphatase 2A and mammalian Target of rapamycin) repeats and a C-terminal kinase domain that has amino acid similarity to phosphatidyl inositol-3 kinase (PI3K) and is flanked and stabilized by conserved FRAP–ATM–TRRAP (FAT) and FAT-C domains. Generation of DSBs and/or changes in chromatin structure lead to activation of ATM and its autophosphorylation on serine 1981 [34]. Activated ATM phosphorylates a multitude of downstream targets including p53, checkpoint kinase 2 (Chk2) and histone H2AX [35]. Indeed, phospho-proteomics studies have identified hundreds of PIKK-dependent, DNA damage-induced phosphorylation events in cells [36,37]. Consistent with its role in the repair of IR-induced DSBs, cell lines with loss or inactivation of ATM are radiation sensitive, have cell cycle checkpoint defects [38] and have defects in slow repair of complex DNA damage lesions and DSBs in the context of heterochromatin [39]. Recently, roles in preventing premature aging [40] and in reactive oxygen sensing [41] have also been reported.

Germline inactivation of both copies of the *ATM* gene causes Ataxia-Telangiectasia (A–T), a devastating childhood condition characterized by ataxia (wobbly gait), telangiectasia (blood vessel abnormalities) and progressive neurodegeneration, particularly in the cerebellum, that renders its victims wheelchair-bound. A–T patients also have immune defects and cancer predisposition and usually succumb to their condition in their early twenties [42]. Accordingly, cell lines derived from A–T patients and ATM knock out mice are hypersensitive to IR and other chemotherapeutic agents [43,44], raising the possibility that cancers with loss of ATM may be more sensitive to DNA damaging agents than their ATM-proficient counterparts [45].

## 3. Targeting ATM-Deficient Cancers

**Figure 1:**
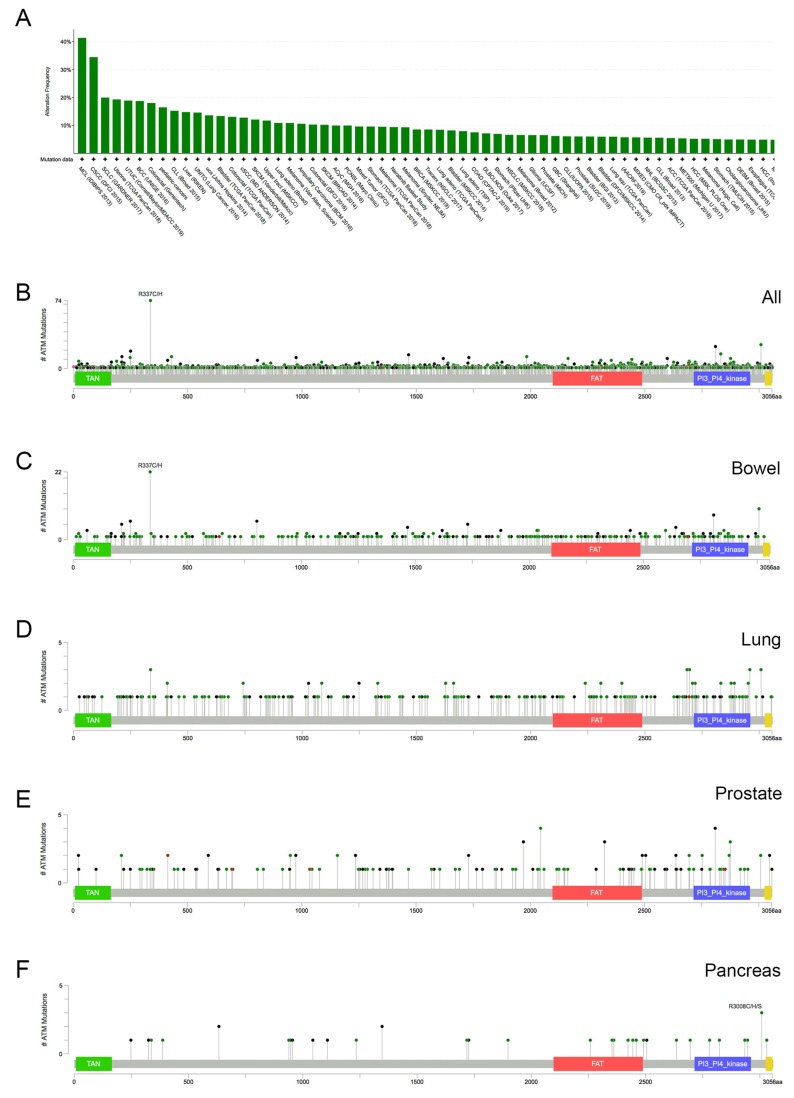
Frequency of Ataxia-Telangiectasia Mutated (*ATM)* mutations in human cancer. (**A**) *ATM* was queried against all entries in the curated non-redundant data set on c-Bioportal (references [46,47]) accessed January 2020. Duplicate studies were removed and copy number variations are not included. The frequency of *ATM* alteration in various cancers is shown. (**B**) ATM is a 3056 amino acid protein consisting of a N-terminal TAN (telomere length maintenance and DNA damage repair) domain (residues 7–165), and a C-terminal kinase domain (residues 2714–2961) flanked by FAT (2097–2488) and FATC (3205–3055) domains. The location of mutations in ATM from all samples in the curated non-redundant data site available on c-Bioportal (references [46,47]), accessed January 2020 (duplicate sets removed and copy number variation not include) is shown. Mutations were distributed across the entire the coding region however, one mutation R337C/H was detected in 74 out of 2263 samples, across all cancers. (**C**) The R337C/H mutation was frequent in bowel cancer (22 out of 331 samples) but less so in lung (panel **D**), prostate (panel **E**) and pancreatic cancers (Panel **F**).

Genome sequencing has revealed that *ATM* is mutated in a variety of human cancers, including mantle cell lymphoma (MCL), colorectal, lung and prostate cancers. Analysis of *ATM* mutation frequency in The Cancer Genome Atlas (TCGA) cohort using c-Bioportal [46,47] indicates that ATM is mutated in approximately 5% of all cancers, with some, such as MCL, with a much higher mutation frequency of ~40% (Figure 1A). Similarly, ATM is mutated in ~20% of colorectal and uterine cancers and approximately 10% of prostate and lung cancers (Figure 1A). The vast majority of these mutations are missense mutations and are scattered throughout the coding region (Figure 1B and [45]). An exception is R377C/H, which occurred in 74 of the 2263 (~3%) of the cancers queried (Figure 1B), and has been identified as a cancer mutation hotspot [48,49]. The R337C/H mutation was prominent in colorectal cancer, but not in prostate, lung or pancreatic cancer (Figure 1C–F). Although the functional consequences of this and most other *ATM* mutations is not known, given that in A–T many mutations in *ATM* induce protein truncation, protein destabilization and resulting loss of function [50], combined with the fact that siRNA-mediated loss of ATM in cancer cell lines results in PARP inhibitor sensitivity [30,31,32], it seems likely that many cancers with *ATM* mutation that lead to loss of function could be candidates for PARP inhibitor treatment.

Given that *ATM* is mutated or lost in over 40% of MCL [51], we and others examined the effects of ATM loss on PARP inhibitor sensitivity in human lymphoma cell lines that lack ATM protein expression. These cell lines were more sensitive to olaparib than their ATM-proficient counterparts in both cell line and animal models [52,53,54]. Moreover, the ATM kinase inhibitor KU55933 enhanced sensitivity to olaparib in ATM-proficient cells indicating that ATM kinase activity protects from PARP inhibitor sensitivity [53]. Similar results were observed in gastric cancer cell lines [55], and in colorectal cancer cell lines with shRNA depletion of ATM [56]. Deletion of ATM in mouse models of lung cancer and pancreatic cancer also induced sensitivity to PARP inhibitors and/or DNA damaging agents, as did inhibitors of the related protein kinase ATR (ATM and Rad3-related) [57,58].

We observed that in MCL, gastric and colorectal cancer cell lines with loss or down regulation of ATM, sensitivity to olaparib was enhanced when *TP53* was also mutated or deleted [52,53,55,56]. However, in mouse models, both *TP53*-proficient and deficient cells were sensitive to olaparib [58]. Therefore, the effect of p53 status on PARP inhibitor sensitivity requires further clarification. Although co-mutation of both *ATM* and *TP53* is rare [59], co-mutation has been shown to occur in 2–3% of non-small cell lung cancer where it increases tumour mutation burden and correlates with better response to immune checkpoint therapy [60], suggesting additional opportunities for targeted therapy for ATM-deficient tumours.

To address the mechanism of olaparib-induced cell sensitivity in human cells lacking ATM, we used CRISPR/Cas9 to delete ATM from the p53-proficient lung adenocarcinoma cell line, A549. In keeping with recent findings, olaparib alone reduced cell proliferation [13], but surprisingly, did not induce cell death [61]. Rather, olaparib was found to induce reversible G2 arrest in ATM-deficient A549 cells [61]. Since the related protein kinase ATR plays a critical role in the G2 checkpoint [62], and given ATM-deficient cells are sensitive to ATR inhibitors [58,63,64,65], we asked whether inhibition of ATR using VE-821 [66,67], would ablate G2 arrest and induce cell death in olaparib-treated ATM-deficient cells. This was indeed found to be the case. Combined treatment with olaparib plus ATR inhibition with VE821 induced cell death only in ATM-deficient A549 cells, suggesting that patients with ATM-deficient tumours could benefit from a combination of PARP and ATR inhibitors [61].

*ATM* is frequently mutated in prostate cancer [68], as well as somatic and hereditary forms of pancreatic cancers [69,70], suggesting that patients with these cancers might also benefit from treatment with a PARP inhibitor. Nevertheless, PROFOUND, a phase III trial that examined the clinical efficacy of olaparib versus standard treatment (abiraterone acetate/enzalutamide) in patients with metastatic, castration-resistant prostate cancer (mCRPC) and HRR gene alterations, revealed less impressive rates of radiographic progression free survival benefit with olaparib in patients with *ATM* alterations, in contrast to patients with other HRR gene alterations (e.g., *BRCA2*), based upon exploratory, hypothesis-generating gene-by-gene subgroup analysis [23]. Thus, it appeared necessary to consider therapeutic approaches that may enhance the efficacy of PARP inhibition in ATM-deficient cancers.

We therefore examined whether the combination of olaparib plus an ATR inhibitor would be effective in cell line models of prostate and pancreatic cancer. We depleted ATM from the prostate cancer cell line PC-3 using CRISPR/Cas9 and found that although olaparib reduced cell proliferation, ATM-deficient cells did not undergo apoptosis unless olaparib was combined with an ATR inhibitor, either VE-821, as in [61] or AZD6738 [71], an ATR inhibitor in clinical trials [72]. Significantly, olaparib and AZD6738 had little effect on ATM-proficient cells either alone or in combination [71]. Similar results were seen with the pancreatic cancer cell line, Panc 10.05, in which ATM was depleted by shRNA [71]. Thus, the combination of PARP and ATR inhibitors could be beneficial in a number of ATM-deficient cancers, including lung, prostate and pancreatic [71]. While A549 has wild type p53 [73], PC-3 are *TP53* null [74] and Panc 10.05 contain a homozygous mutation at I225N [75,76], therefore these data suggest that the sensitivity of ATM-deficient cells to the combination of PARP inhibitor and ATR inhibitor is not dependent on p53 status.

Our results also highlight the importance of the method used to assess cell viability in determining sensitivity of a cell line to a particular agent. Although ATM-deficient cells were highly sensitive to olaparib in clonogenic survival assays and the number of viable cells was decreased compared to ATM-proficient cells measured using the trypan blue exclusion assay, analysis of sub-G1 DNA or annexin staining did not reveal evidence of cell death [61,71]. Rather, olaparib-treated, ATM-deficient cells underwent reversible G2 arrest, and did not undergo cell death until an ATR inhibitor was also added [61,71]. Moreover, similar results have been seen in a patient-derived xenograft model of *BRCA*-mutant high-grade serous ovarian cancer, suggesting that PARP inhibition also increases reliance on ATR-dependent G2 arrest in BRCA-deficient cells [77], thus the combination of PARP and ATR inhibition may have benefits in other HDR-deficient cancers.

## 4. ATM Mutation Versus Loss of Function: Identifying Patients Who May Benefit from PARP Inhibitor Treatment

To date, most work from our lab and others has centred on the effects of olaparib on cell lines or mice in which ATM has been deleted [52,53,54,57,58,61,71] or inhibited with KU55933 [53,55]. An important difference is that in cancer, ATM is mutated, but the effects of these mutations on ATM function are, for the most part, unknown. As shown in Figure 1, literally hundreds of mutations have been identified in ATM and these mutations are scattered throughout the coding region. Apart from R337H/C, a hotspot mutation prevalent in colorectal cancer, other individual mutations are seen less frequently and their effects on ATM function is not known. Recently, three papers describe cryo-electron microscopy structures of Tel1, the well-conserved ATM homolog in lower eukaryotes, showing that ATM forms an autoinhibited dimer [78,79,80], providing insight into the conformational changes necessary for ATM activation. Three of these structures provide atomic models of the C-terminal kinase domain, and one, from a thermophilic fungus, also provides an atomic model of the majority of the N-terminal heat repeat domain in open and closed conformations. These structures provide the molecular basis to begin to understand cancer-associated *ATM* mutations. Initial analyses predict many cancer-associated mutations in the kinase domain are likely to impact ATM activity or protein folding [78,79], and the equivalent residue to R337 appears to stabilize the packing of two helices in the N-terminal domain, with the R337C/H mutation possibly destabilizing this region of ATM [78]. The methods developed to purify wildtype ATM homologs pave the way for more comprehensive studies to test the effect of cancer-associated mutations on ATM activities and stability.

For the treatment of cancer patients based on *ATM* status, it will be critical to identify those patients who have mutations that impact ATM activity or stability and are therefore most likely to benefit from PARP/ATR inhibitor combination treatment. This may be challenging by DNA sequencing alone, given the number of mutations identified and that their effects on ATM function are, for the most part, not known. That being said, preliminary clinical trials have shown promising, if not controversial results. In an initial trial of 60 men with mCRPC, 5 were shown to have mutation of *ATM* and 4 responded to olaparib [81]. A subsequent larger trial in a similar patient population demonstrated 7 of 19 patients with ATM mutation met at least one response criteria [82]. In contrast, a multicentre retrospective review of patients with mCRPC treated with olaparib showed no patients with *ATM* mutations achieved a PSA response and had significantly worse progression-free survival (PFS) and overall survival compared to *BRCA*-mutated patients. The addition of olaparib to paclitaxel failed to improve overall survival over paclitaxel alone in Asian patients with recurrent gastric cancer, both in the overall patient population as well as those with low or absent ATM expression, as determined by immunohistochemistry (IHC) [83]. Our findings that olaparib alone is cytostatic in ATM-deficient cells and that PARP and ATR inhibitors need to be combined to kill ATM-deficient cells is in line with these findings and suggest that this combinatorial approach could improve long-term survival in patients.

**Figure 2:**
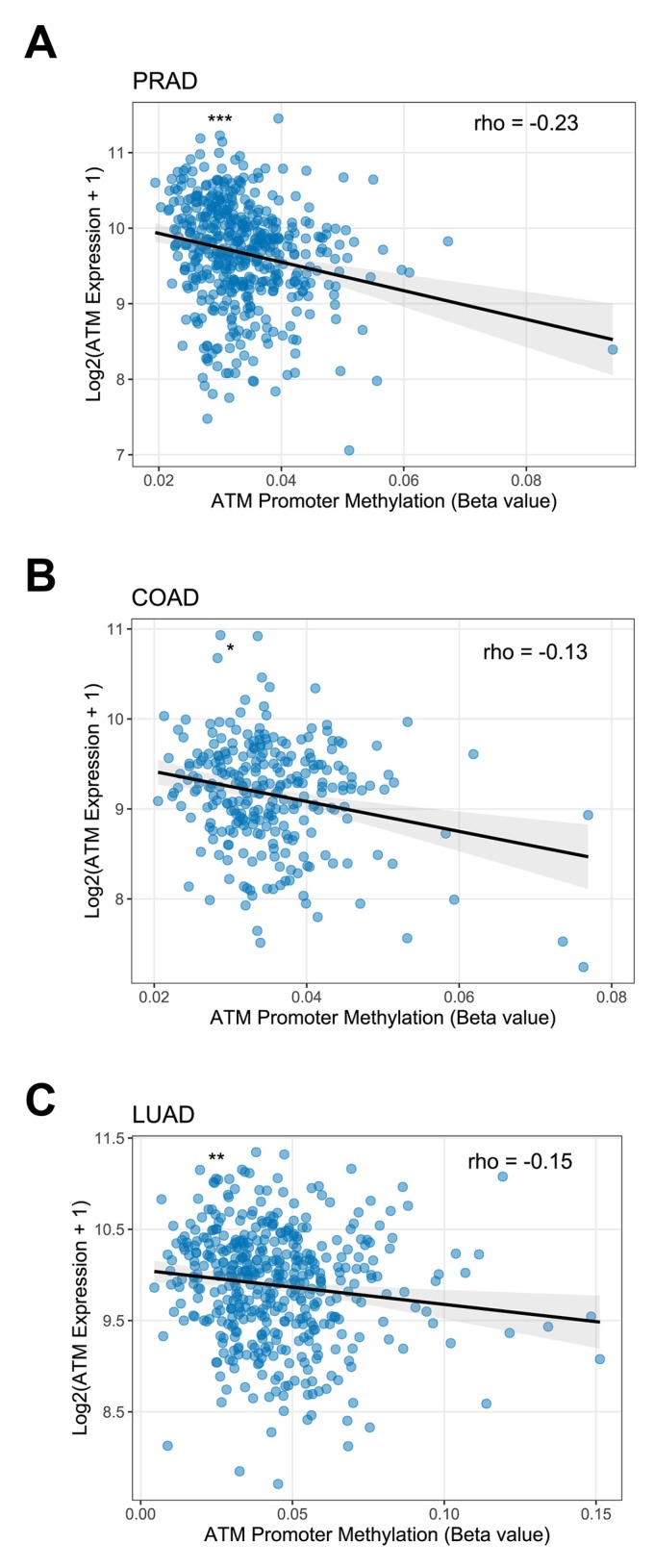
*ATM* promoter methylation and *ATM* gene expression in adenocarcinomas. Scatter plots showing the correlation between methylation beta values of the *ATM* promoter probe cg01756564 and *ATM* gene expression in TCGA datasets of (**A**) prostate adenocarcinoma (PRAD, *n* = 496), (**B**) colon adenocarcinoma (COAD, *n* = 276) and (**C**) lung adenocarcinoma (LUAD, *n* = 454). Spearman’s rho values are indicated in the top right. Grey region of linear fit indicates 95% confidence interval. Asterisks in the top left indicate significance. * = *p* < 0.05, ** = *p* <0.01, *** = *p* < 0.001.

Despite the large number of uncharacterized mutations in *ATM* in cancer, many mutations known to impact ATM function, such as those resulting in A–T, frequently induce protein destabilization [50]. Therefore, determining the presence of ATM protein in cancer patient biopsy samples through IHC or other approaches may prove useful. Interestingly, although data from TCGA reveals that the *ATM* gene is mutated in 12–14% of patient samples with lung adenocarcinoma, another study has reported that ~40% of lung adenocarcinoma patients have low ATM protein expression by IHC [84]. A possible explanation for the apparent difference between *ATM* gene alteration and ATM protein expression could be methylation of the *ATM* promoter leading to transcription silencing [85]. Indeed, our analysis of methylation data in TCGA datasets revealed significant negative correlations between *ATM* promoter methylation and *ATM* gene expression in prostate adenocarcinoma (*p* = 1.962 × 10^−7^, Spearman’s rho = −0.23), lung adenocarcinoma (*p* = 0.001159, Spearman’s rho = −0.15) and colon adenocarcinoma (*p* = 0.0372, Spearman’s rho = −0.13) (Figure 2). These findings suggest that ATM promoter methylation may play a role in the regulation of *ATM* gene expression in these cancer types.

Another possibility for stratifying patients who will benefit from a combination of PARP and ATR inhibitors would be to use a surrogate marker of ATM functionality, either through the use of phosphospecific antibodies to ATM itself or its downstream targets or an RNA signature specific to loss of ATM functionality. Indeed, the search for identifying tumours which exhibit “BRCAness” or HRR deficiency is an area of active investigation [27,86]. Answers to these questions and more may become apparent over the next few years as PARP inhibitor therapy is tested in more patients with defects in DNA damage response genes.

## 5. Concluding Remarks

In conclusion, basic research into DNA damage repair biochemistry led to the identification of PARP, the inhibition of which, almost 40 years later, is showing great promise in the treatment of *BRCA*-deficient ovarian, breast, prostate and pancreatic cancer patients [87,88,89]. Work from our lab and others discussed here has shown that PARP inhibitors may also have potential in treating patients with ATM-deficient tumours. Our recent studies have revealed that in ATM-deficient cancer cell lines, olaparib is cytostatic not cytotoxic and that combination of olaparib with an ATR inhibitor is needed to induce cell death [61,71]. Moreover, similar results were seen in models of BRCA-mutant ovarian cancer, suggesting that inhibition of ATR potentiates the effects PARP inhibition in *BRCA*-deficient cells [77], thus the combination of PARP and ATR inhibition may have benefits in other HDR-deficient cancers. It will be exciting to see whether this finding will also apply to other PARP inhibitors and, most importantly, in the clinic for improving outcomes for cancer patients. Indeed, several clinical trials using a PARP inhibitor in combination with an ATR inhibitor are ongoing (Table 1).

It is also possible that ongoing preclinical studies will reveal new synthetic lethal interactions within the DNA damage response. Indeed, inhibitors to other proteins in the DNA damage response are being developed [27]. Identification of alternative DNA damage response genes to target in cancer could be useful as therapies in their own right and also in cases where tumours become resistant to other therapies, such as is currently observed in *BRCA*-deficient tumours treated with PARP inhibitors [90,91]. Finally, ATM has widespread cellular roles outside the DNA damage response, including roles in cellular redox signalling [41] and regulation of autophagy [92,93], apoptosis and other cell death pathways [94,95,96,97,98], further increasing potential opportunities to target ATM-deficient cells with novel therapies.

## Figures and Tables

**Table 1:** List of ongoing clinical trials combining a poly-ADP ribose polymerase (PARP) inhibitor with an ATR inhibitor. Information obtained from https://clinicaltrials.gov, accessed March 9 2020.

Clinical Trial Number	PARP Inhibitor	ATR Inhibitor	Other Therapy/Status	Cancer Type
NCT02723864	Veliparib/ABT-888	VX-970	Cisplatin	Refractory Solid Tumours
NCT034R2342	Olaparib	AZD6738	Platinum-sensitive or platinum-resistant	Recurrent ovarian cancer (CAPRI trial)
NCT03682289	Olaparib	AZD6738	None stated	Renal cell carcinoma, urothelial carcinoma, pancreatic cancers and other solid tumours
NCT03787680	Olaparib	AZD6738	DNA repair proficient/DNA repair deficient	Metastatic Castration-Resistant Prostate Cancer (TRAP trial)
NCT04065269	Olaparib	AZD6738	ARID1A loss versus no loss	Relapsed gynaecological cancers
NCT04267939	Niraparib	BAY1895344		Recurrent Advanced Solid Tumours and Ovarian Cancer
